# Molecular detection and clinicopathological characteristics of advanced/recurrent biliary tract carcinomas harboring the *FGFR2* rearrangements: a prospective observational study (PRELUDE Study)

**DOI:** 10.1007/s00535-020-01735-2

**Published:** 2020-10-26

**Authors:** Yuta Maruki, Chigusa Morizane, Yasuhito Arai, Masafumi Ikeda, Makoto Ueno, Tatsuya Ioka, Atsushi Naganuma, Masayuki Furukawa, Nobumasa Mizuno, Tadashi Uwagawa, Naminatsu Takahara, Masashi Kanai, Akinori Asagi, Satoshi Shimizu, Atsushi Miyamoto, Seigo Yukisawa, Makoto Kadokura, Yasushi Kojima, Junji Furuse, Takako Eguchi Nakajima, Kentaro Sudo, Noritoshi Kobayashi, Natsuko Hama, Takeharu Yamanaka, Tatsuhiro Shibata, Takuji Okusaka

**Affiliations:** 1 Department of Hepatobiliary and Pancreatic Oncology, National Cancer Center Hospital, 5-1-1 Tsukiji, Chuo-ku, Tokyo, 104-0045 Japan; 2 Division of Cancer Genomics, National Cancer Center Research Institute, Tokyo, Japan; 3 Department of Hepatobiliary and Pancreatic Oncology, National Cancer Center Hospital East, Kashiwa, Japan; 4 Department of Gastroenterology, Hepatobiliary and Pancreatic Medical Oncology Division, Kanagawa Cancer Center, Kanagawa, Japan; 5 Department of Oncology Center, Yamaguchi University Hospital, Yamaguchi, Japan; 6 Department of Gastroenterology, National Hospital Organization Takasaki General Medical Center, Gunma, Japan; 7 Department of Hepato-Biliary-Pancreatology, National Hospital Organization Kyushu Cancer Center, Fukuoka, Japan; 8 Department of Gastroenterology, Aichi Cancer Center Hospital, Aichi, Japan; 9 Department of Surgery, The Jikei University School of Medicine, Tokyo, Japan; 10 Department of Gastroenterology, Graduate School of Medicine, The University of Tokyo, Tokyo, Japan; 11 Department of Therapeutic Oncology, Graduate School of Medicine, Kyoto University, Kyoto, Japan; 12 Department of Gastrointestinal Medical Oncology, National Hospital Organization Shikoku Cancer Center, Ehime, Japan; 13 Department of Gastroenterology, Saitama Cancer Center, Saitama, Japan; 14 Department of Surgery, National Hospital Organization Osaka National Hospital, Osaka, Japan; 15 Department of Medical Oncology, Tochigi Cancer Center, Tochigi, Japan; 16 Department of Gastroenterology, Kofu Municipal Hospital, Yamanashi, Japan; 17 Department of Gastroenterology, National Center for Global Health and Medicine, Tokyo, Japan; 18 Department of Medical Oncology, Kyorin University Faculty of Medicine, Tokyo, Japan; 19 Department of Clinical Oncology, St.Marianna University School of Medicine, Kanagawa, Japan; 20 Kyoto Innovation Center for Next Generation Clinical Trials and iPS Cell Therapy, Kyoto University Hospital, Kyoto, Japan; 21 Division of Gastroenterology, Chiba Cancer Center, Chiba, Japan; 22 Department of Oncology Division, Yokohama City University School of Medicine, Kanagawa, Japan; 23 Department of Biostatistics, Yokohama City University Graduate School of Medicine, Kanagawa, Japan

**Keywords:** Advanced/recurrent biliary tract cancer, *FGFR2* rearrangement, Fluorescent in situ hybridization, RNA sequencing

## Abstract

**Background:**

*Fibroblast growth factor receptor 2* (*FGFR2*) rearrangement is expected to be a novel therapeutic target in advanced/recurrent biliary tract cancer (BTC). However, efficient detection and the exact frequency of *FGFR2* rearrangements among patients with advanced/recurrent BTC have not been determined, and the clinical characteristics of *FGFR2* rearrangement-positive patients have not been fully elucidated. We aimed to determine the frequency of *FGFR2* rearrangement-positive patients among those with advanced/recurrent BTC and elucidate their clinicopathological characteristics.

**Methods:**

Paraffin-embedded tumor samples from formalin-fixed surgical or biopsy specimens of patients with advanced/recurrent BTC were analyzed for positivity of *FGFR2* rearrangement by fluorescent in situ hybridization (FISH). RNA sequencing was performed on samples from all FISH-positive and part of FISH-negative patients.

**Results:**

A total of 445 patients were enrolled. FISH was performed on 423 patients (272 patients with intrahepatic cholangiocarcinoma (ICC), 83 patients with perihilar cholangiocarcinoma (PCC), and 68 patients with other BTC). Twenty-one patients with ICC and four patients with PCC were diagnosed as *FGFR2*-FISH positive. Twenty-three of the 25 FISH-positive patients (20 ICC and 3 PCC) were recognized as *FGFR2* rearrangement positive by targeted RNA sequencing. Younger age (≤ 65 years; *p* = 0.018) and HCV Ab- and/or HBs Ag-positivity (*p* = 0.037) were significantly associated with the presence of *FGFR2* rearrangement (logistic regression).

**Conclusions:**

*FGFR2* rearrangement was identified in ICC and PCC patients, and was associated with younger age and history of hepatitis viral infection.

**Electronic supplementary material:**

The online version of this article (10.1007/s00535-020-01735-2) contains supplementary material, which is available to authorized users.

## Introduction

Patients with biliary tract cancer (BTC) have a poor prognosis, with a 5 year survival rate of 22.5% [1]. BTC consists of intrahepatic cholangiocarcinoma (ICC), perihilar cholangiocarcinoma (PCC), gallbladder carcinoma (GBC), distal cholangiocarcinoma (DCC), and ampullary carcinoma (AC), and the biological characteristics and prognosis vary depending on the primary site [2].

The international standard first-line chemotherapy for advanced/recurrent BTC is gemcitabine plus cisplatin therapy; however, the median overall survival is only 11.7 months in the ABC-02 study, which established this therapy as its standard [3].

Genomic analyses of BTC have led to the development of molecular target therapy [4]. Especially, *isocitrate dehydrogenase *(*IDH*) mutations [5, 6] and *fibroblast growth factor receptor 2 *(*FGFR2*) fusion genes [7–12] in ICC have been identified as important driver alterations and are promising therapeutic targets. Actually, the US Food and Drug Administration (FDA) granted accelerated approval to pemigatinib for cholangiocarcinoma with an *FGFR2* rearrangement or fusion in April 2020 based on the favorable results of a clinical trial [13].

Fibroblast growth factor/fibroblast growth factor receptor (FGF/FGFR) signaling plays a role in the development of normal organs and blood vessels, as well as in skeleton formation. *FGFR* rearrangements (fusions/truncations) autonomously activate the FGF signaling pathway and are involved in breast cancer, lung cancer, gastric cancer, and hematological tumors [10]. *FGFR2* rearrangements are considered to be one of the important driver genes in ICC, with 9–14% of ICC cases reported as positive for *FGFR2* rearrangements [11–14].

Several clinical trials targeting *FGFR2* rearrangements have already been conducted [8, 9, 13], but there is only limited information of its positivity rate and the related clinical features in advanced/recurrent cases. In addition, although the positive rate of *FGFR2* rearrangements has been reported in ICC, data are not available for the other BTCs. Thus, to verify whether, in addition to ICC, other BTCs are also associated with *FGFR2* rearrangements, investigations should be extended to all advanced/recurrent BTCs, including those outside of ICC.

Various molecular diagnostic methods can be applied for the detection of the oncogenic fusion genes such as *FGFR2* and *ALK*, i.e., multiplex RT-PCR, fluorescent in situ hybridization (FISH) and target-panel DNA sequencing or RNA sequencing from frozen or formalin-fixed paraffin-embedded (FFPE) specimens. However, more than 40 genes have been identified as fusion partner with *FGFR2*, but comprehensive identification of fusion genes takes time and often becomes a problem in medical practice. Among those candidates, we adopted FISH assay for screening *FGFR2* rearrangements in this prospective study, because the majority of our cases were expected not to have archival surgical tissue and it is frequently difficult to obtain enough volume of tumor tissue for many tests including next-generation sequencing (NGS). FISH is also a highly preferable method for oncogenic gene rearrangement detection in the point of short turnaround time, low cost, and requiring smaller amounts of tissue (than NGS) [15]. We also performed post hoc RNA sequencing and validated the FISH results in this study.

## Methods

### Study design and patients

This was a prospective observational multicenter study conducted in Japan. The subjects were patients with advanced/recurrent BTC (ICC, PCC, DCC, GBC, and AC), histologically confirmed as adenocarcinoma or adenosquamous carcinoma, who were scheduled for or had received systemic chemotherapy. FISH analysis was performed for all BTCs between March 2014 and February 2016 (the first period). Since, in this period, positive patients were only found among those with ICC and PCC, only patients with these two BTCs were enrolled in the subsequent study period between October 2016 and November 2018.

This study was approved by the institutional review boards from all participating institutions. Written consent was obtained directly from patients, and the study was conducted in accordance with the Declaration of Helsinki, the “Ethical Guidelines for Epidemiology Research,” and the “Ethical Guidelines for Medical and Health Research Involving Human Subjects.” This trial was registered in UMIN Clinical Trials Registry (UMIN-CTR) under the registration number UMIN000014767.

### Sample preparation

For efficient and prospective diagnosis of the status of *FGFR2* rearrangement, we performed the break-apart FISH assays using paraffin-embedded tumor samples from formalin-fixed surgical or biopsy specimens as described below. After patients were enrolled, one hematoxylin and eosin (HE) stained, and five unstained sections, of 4 µm thickness, were prepared from the FFPE samples. The HE-stained section was used for marking tumors, while FISH analysis and targeted RNA sequencing were performed using one and two unstained sections, respectively.

To set the cutoff value for the FISH analysis, we used previously reported *FGFR2* fusion-positive and -negative cases from surgically resected specimens [16]. Three *FGFR2* fusion-positive cases and nine negative cases validated by RNA sequencing were used in the assay. In fusion-positive cases, two showed YGR FISH signal (80% and 91%) and one showed YR FISH signal (88%). Fusion-negative cases showed background FISH signals at 6.4% (mean + 2SD). From these results, we initially defined the cutoff value of ≥ 7% for the positive cells in the FISH analysis. We also performed RNA sequencing, targeting 1385 genes including *FGFR2*, for all 197 cases evaluated by FISH in the first period except 16 cases with low RNA-quantity specimens, and on 18 FISH-positive cases in the second period.

We collected clinical information of the patients who got FISH analyses. The following items were considered at enrollment: age, sex, family history, occupational history of working in the printing industry, disease status, degree of histological differentiation, macroscopic type, smoking history, drinking history, HCV antibody, HBs antigen, cholelithiasis, past history of primary sclerosing cholangitis (PSC), and presence/absence of pancreaticobiliary maljunction. In the second period, the survival of *FGFR2* rearrangement-positive patients and the use of FGFR inhibitors were also investigated.

The primary end point was the frequency of *FGFR2* rearrangement-positive patients among those with BTC. The secondary end point was the correlation between the presence of *FGFR2* rearrangement and the clinical characteristics of the patients.

### FISH analysis

**Fig. 1:**
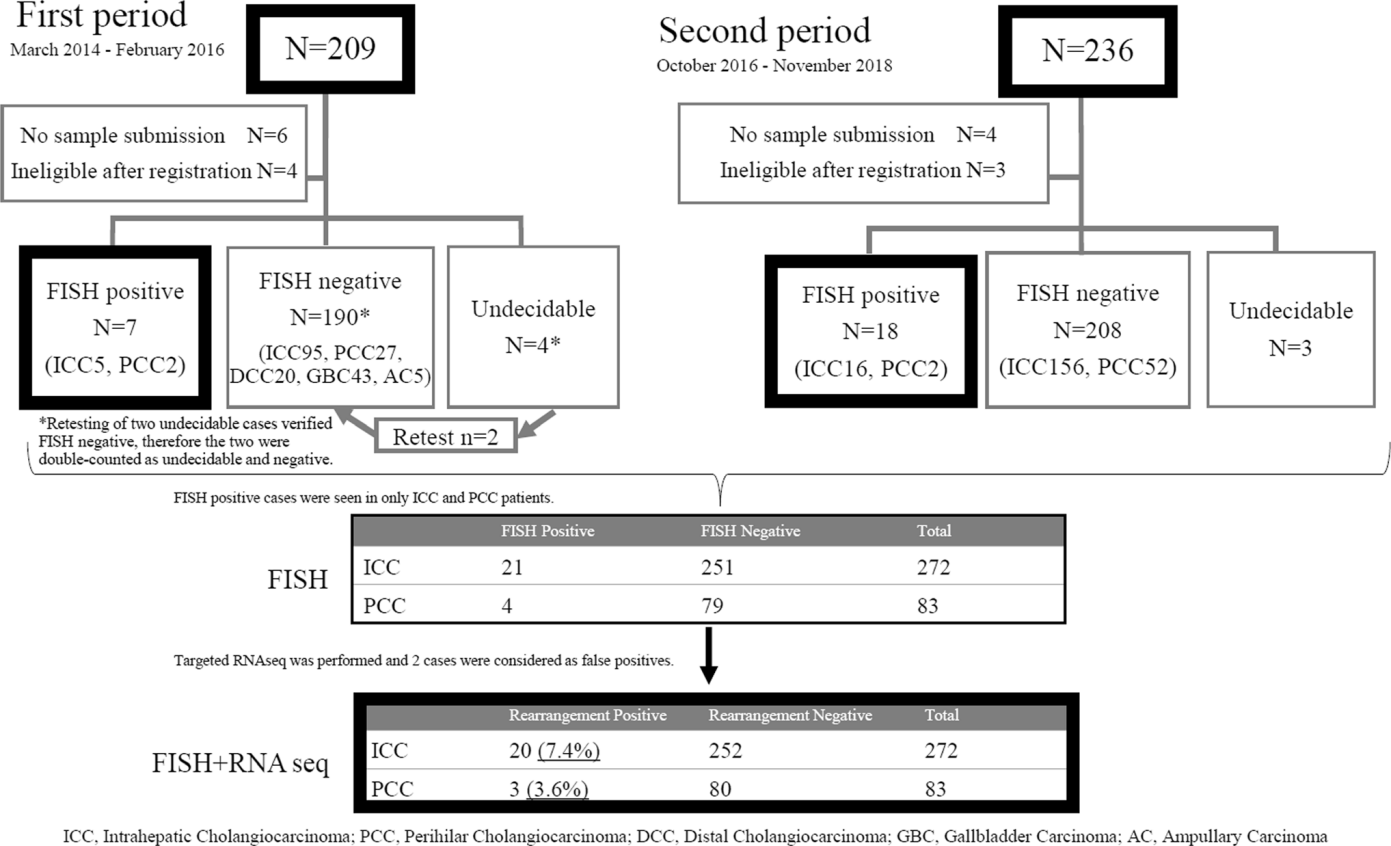
Study flow and summary of *FGFR2* rearrangement detection

To identify *FGFR2* rearrangements, break-apart FISH assays were performed on FFPE tumors using a probe set, which hybridizes with the neighboring 5’-telomeric (RP11-78A18, labeled with Spectrum Green) and 3’-centromeric (RP11-7P17, labeled with Spectrum Red) sequences of the *FGFR2* gene (Chromosome Science Labo Inc., Sapporo, Japan). One-hundred non-overlapping tumor cells with at least one 5’ and one 3’ signal, whether fused or separated, were examined and a detailed signal pattern was recorded at a clinical laboratory testing company with a turnaround time between 7 and 10 days (LSI medience, Tokyo, Japan). A fused 5’/3’ signal may appear yellow due to co-localization of green (5’ probe) and red (3’ probe) signals. A split signal was defined by 5’ and 3’ probes observed at a distance > 1 time the signal size, and signals separated by less than this distance were regarded as fused signals. The rearrangement-positive cells were defined as having any split signal (YGR FISH type) or isolated green signal (YG type), while any isolated red signal (YR type) was treated as rearrangement-negative, because this type denotes 5’ probe deletion of *FGFR2* (Supple Fig. 1). The rate of rearrangement-positive cells was calculated for each case. These scoring criteria were developed and validated internally by using genotyped positive and negative controls from surgically resected specimens.

### Targeted RNA sequencing

Total RNA was isolated from one or two FFPE tumor sections with 4 µm thickness using an miRNeasy FFPE kit (Qiagen, Hilden, Germany). The quantity of the RNA was determined with a NanoDrop instrument (ThermoFisher, Waltham, MA, USA). A targeted RNA sequencing library was prepared from 25–200 ng total RNA using a TruSight RNA PanCancer library kit (Illumina, San Diego, USA), which covers 1385 cancer-related genes including *FGFR2*. The library was subjected to paired-end sequencing of 151-bp fragments on a MiSeq DNA sequencer (Illumina). We obtained at least 50 million reads per sample, and the paired-end reads were mapped and aligned to known RNA sequences in the RefSeq, Ensembl, and LincRNA databases with the BWA-MEM program. After selecting the best hits with the proper spacing and orientation, gene expression values were calculated as reads per kilobase of exon per million mapped reads (RPKM). *FGFR2* gene expression is indicated as the ratio (fold) of RPKM between each sample and the median value of FISH-negative cases (RPKM = 162.9). Aberrant paired reads that mapped to different transcription units of *FGFR2* were identified as *FGFR2* rearrangements. Junction reads revealed in-frame gene fusions between *FGFR2* and other genes. In six cases (case nos. 2, 3, 4, 5, 24 and 25), another targeted RNA sequencing method of Anchored Multiplex PCR assay to detect *FGFR* fusions (AMP-FGFR), FusionPlex FGFR panel (Archer DX, Boulder, USA) was performed. Using 50 ng total RNA, sequencing libraries of targeting *FGFR* genes (*FGFR1, 2* and *3*) were sequenced and analyzed according to the manufacturer’s protocols.

### Statistical analysis

The primary objective was to estimate the rate of *FGFR2* rearrangement-positive patients. With an expected positive *FGFR2* rearrangement rate of 20–40%, a total of 100 patients were planned for achieving a target width of less than 10% for the two-sided 95% confidence interval. The correlations between the presence of *FGFR2* rearrangement and the clinical characteristics of the patients were analyzed using the Fisher’s exact test and logistic regression. All tests were two-sided, and a *p* value < 0.05 was considered statistically significant. Younger age was defined under the median age of 65 years, and excessive alcohol abuse was defined as consumption of 60 g or more of pure ethanol per day according to the WHO criteria. The *FGFR2* rearrangement-positive patients enrolled in the second period were included in the survival analysis. The starting date for the overall survival analysis was the date of first-line treatment initiation, and the ending date was the date on which an event was censored, or death occurred. Survival was analyzed using the Kaplan–Meier method. The software used for analyses was SPSS 22.0 (IBM Corp. SPSS Inc., Armonk, NY, USA).

## Results

### Study flow

A total of 445 Japanese patients were enrolled from 20 institutions for this prospective study, and the analysis diagram is summarized in Fig. 1. There were seven patients in whom the FISH assay was undecidable (two cases were successfully reported in retest) due to no signal (potentially insufficient sample amount) in three cases, self-luminous primarily due to inappropriate marking (ink containing a fluorescence) during formation of the paraffin block in three cases, and peeling off of the paraffin section due to unsuitable slide glass lacking silane coating in one case. Results of the FISH assay were successfully reported on 423 patients (Suppl Fig. 1, 2). Table 1 shows the clinical characteristics of the patients. Regarding the primary sites, 64% and 20% of patients were ICC and PCC, respectively. The mean time between enrollment and FISH report delivery was 13 days (7–31 days). There are two periods of patient enrollment in this study, where 209 patients were in the first period and 236 patients were in the second period. Four patients in the first period and three in the second period failed to be analyzed by the FISH assay, and two patients in the first period were retested using a different tumor tissue with negative results obtained. The FISH assay was performed a total of 432 times, yielding 425 results (98.3%). The success rate of the FISH assay in the biopsy specimen and surgically resected specimens was 97.5% (273/280) and 100% (152/152), respectively.

**Table 1:** Clinical characteristics of the study patients (*N* = 423)

Characteristic	*N* (%)
Sex	
Male	280 (66)
Female	143 (34)
Age	
> 65	191 (45)
≤ 65	232 (55)
Tested tumor specimen	
Resection	152 (36)
Biopsy	271 (64)
Stage	
Metastasis	221 (52)
Recurrence after resection	108 (26)
Locally advanced	94 (22)
Differentiation	
Poorly differentiated	75 (18)
Moderate/well differentiated	166 (39)
Unknown	182 (43)
Primary site	
ICC	272 (64)
ECC	
PCC	83 (20)
DCC	20 (5)
GBC	43 (10)
AC	5 (1)

*ICC* intrahepatic cholangiocarcinoma, *ECC* extrahepatic cholangiocarcinoma, *PCC* perihilar cholangiocarcinoma, *DCC* distal cholangiocarcinoma, *GBC* gallbladder carcinoma, *AC* ampullary carcinoma

### FISH analysis

Of the 423 patients, 25 were diagnosed as *FGFR2* rearrangement positive (*FGFR2*-FISH positive) using the proper FISH pattern criteria (YGR or YG, except YR), and the ≥ 7% cutoff value (Supple Fig. 1). The number of *FGFR2*-FISH positives was 21/272 cases and 4/83 cases among patients with ICC and PCC, respectively (Fig. 1).

### RNA sequencing

RNA sequencing targeting 1385 genes including *FGFR2* was performed on the 25 patients determined to be *FGFR2*-FISH positive, and in-frame *FGFR2* fusion transcripts were detected in 19 cases (Table 2). Fourteen kinds of *FGFR2* fusion partner genes were identified, and *FGFR2-BICC1* was frequently detected (4/19). However, no *FGFR2* fusion transcript was detected in the remaining six *FGFR2*-FISH-positive cases. One of the cases that was *FGFR2*-FISH positive and fusion transcript negative showed an YGR (split) FISH pattern; meanwhile the other five showed a YG (loss of 3’ probe) FISH pattern, with relatively low *FGFR2* gene expression ratio (1.50 – 2.59) observed, except for one case (case 6, 7.56). In other two cases showing the YG pattern in FISH assay, *FGFR2* fusion transcripts were detected as *FGFR2-CCDC6* and *FGFR2-BICC1*, respectively. Moreover, the *FGFR2* gene expression was high (5.10 and 18.33) in these two cases. Discrimination of *FGFR2* fusion transcript positive or negative was validated in six cases (cases 2, 3, 4, 5, 24 and 25) by another RNA sequencing method, AMP-FGFR, targeting *FGFR1, FGFR2* and *FGFR3* genes, and the results of the identified *FGFR2* fusion transcripts or no *FGFR2* fusion transcripts were coincident completely with the prior ones. In two fusion transcript-negative cases (case 24, 25), the FISH-positive cell rates (8%, 9%) were close to the ≥ 7% preset cutoff value, causing us to treat them as false positives after RNA sequencing. In the remaining 190 *FGFR2*-FISH-negative cases from the first period, RNA sequencing targeting 1385 genes was also performed on all cases, except 16 with low RNA quantity, to survey the false negative cases in the FISH analysis. Results show that all cases were confirmed to be negative for *FGFR2* fusion transcripts. This implies no false negative cases in the FISH assay for *FGFR2* rearrangement. Taken together, 23 patients were identified as *FGFR2* rearrangement positive including 20 (7.4%) ICC cases and three (3.6%) PCC cases.

**Table 2:** Details of all *FGFR2* rearrangement-positive cases

Case	Sex	Age	Stage	Primary site	HBV/ HCV^a^	History of smoking	Alcohol^b^	FGFR TKI^c^	OS (days)	FISH + cell rate (%)	FISH type	Targeted RNAseq	FGFR2 expression (fold)^d^
1	F	57	Recurrence	PCC	+	Y	N	na	na	94	YGR	*FGFR2-POC1B*	1.7
2	M	73	Metastatic	ICC	−	Y	N	na	na	89	YG	No fusion	1.5
3	F	75	Metastatic	ICC	−	N	N	na	na	90	YGR	*FGFR2-PAWR*	8.0
4	M	46	Locally advanced	ICC	−	Y	N	na	na	87	YGR	*FGFR2-AHCYL1*	4.0
5	M	53	Metastatic	ICC	−	Y	Y	na	na	78	YGR	*FGFR2-SHC1*	10.9
6	M	60	Metastatic	ICC	−	N	N	−	1547	24	YG	No fusion	7.6
7	M	52	Metastatic	ICC	+	Y	N	+	1081	90	YGR	*FGFR2-BICC1*	27.1
8	M	61	Recurrence	PCC	−	N	N	+	462	15	YG	No fusion	2.6
9	M	69	Metastatic	ICC	+	Y	N	−	420^†^	91	YGR	*FGFR2-ZMYM4*	2.3
10	M	65	Recurrence	ICC	−	Y	N	+	551	87	YGR	*FGFR2-SLMAP*	1.7
11	M	41	Recurrence	ICC	−	N	N	+	716	87	YG	*FGFR2-CCDC6*	5.1
12	M	59	Metastatic	ICC	+	Y	Y	+	1166^†^	69	YGR	*FGFR2-BICC1*	5.9
13	M	66	Metastatic	ICC	−	Y	N	+	920^†^	93	YGR	*FGFR2-BICC1*	5.7
14	F	57	Metastatic	ICC	+	Y	N	+	662	94	YGR	*FGFR2-TRIM55*	2.2
15	F	40	Metastatic	ICC	−	Y	Y	+	430	89	YGR	*FGFR2-POC1B*	3.2
16	M	59	Metastatic	ICC	−	Y	N	+	676^†^	85	YGR	*FGFR2-SLMAP*	1.5
17	M	53	Metastatic	ICC	−	N	Y	+	1304	41	YGR	*FGFR2-SMARCC1*	5.4
18	M	62	Recurrence	ICC	+	Y	N	+	453	89	YGR	*FGFR2-AFF3*	4.5
19	M	43	Recurrence	ICC	−	Y	Y	+	804	91	YGR	*FGFR2-HOOK1*	5.5
20	M	66	Recurrence	ICC	−	Y	Y	+	559	91	YG	*FGFR2-BICC1*	18.3
21	M	62	Metastatic	ICC	−	Y	Y	−	231	93	YGR	*FGFR2-SHROOM3*	15.4
22	M	65	Recurrence	ICC	−	N	N	−	300^†^	85	YGR	*FGFR2-MYH9*	6.8
23	M	66	Locally advanced	PCC	−	Y	N	−	364	94	YG	No fusion	2.0
24	F	64	Metastatic	PCC	−	Y	N	na	na	8	YG	No fusion	1.8
25	F	39	Metastatic	ICC	+	N	N	na	na	9 (88)	YGR (YR)	No fusion	1.8

*M* male, *F* female, *ICC* intrahepatic cholangiocarcinoma, *PCC* perihilar cholangiocarcinoma, *Y* smoker or drinker, *N* non-smoker or non-drinker, *OS* overall survival, *FGFR TKI* FGFR tyrosine kinase inhibitor, *RPKM* reads per kilobase of exon per million mapped reads, *na* no available data

^a^HBV or HCV hepatitis virus infection positive ( + ) or negative ( − ) was analyzed with HBs antigen and HCV antibody

^b^Drinker is defined as drinking equal or more than 60 g of ethanol a day

^c^Treatment with FGFR-TKI was performed ( + ) or not ( − )

^d^FGFR2 gene expression is indicated as the ratio (fold) of RPKM between each sample and the median value of FISH-negative cases

^†^ dead

### Clinical features and prognosis

The clinical characteristics were evaluated in the 23 cases with *FGFR2* rearrangements. The macroscopic type of *FGFR2* rearrangement-positive ICC was invariably the mass forming type (Table 3). Univariate analysis unveiled associations between the presence of *FGFR2* rearrangement and two factors, i.e., younger age (≤ 65 years; *p* = 0.0085), and HCV Ab- and/or HBs Ag-positivity (*p* = 0.02). Although a history of heavy drinking (ethanol ≥ 60 g/day) also tended to be associated with positivity for *FGFR2* rearrangement, no statistically significant difference was achieved (*p* = 0.06). Furthermore, multivariate analysis of these three factors identified younger age (≤ 65 years) and HCV Ab- and/or HBs Ag-positivity as associated with *FGFR2* rearrangement (Table 4). The median age of *FGFR2* rearrangement positive/negative patients was 60 years old (range 40–75)/67 years old (range 25–91), respectively. Thus, 10.3% (17/165) of younger patients and 16.6% (6/36) of hepatitis virus-positive patients were *FGFR2* rearrangement positive. Of the patients who were positive for both factors, 26% showed *FGFR2* rearrangements.

**Table 3:** Univariate analysis of *FGFR2*-positive patients determined by FISH and RNAseq results (*N *= 355). The analysis was limited to ICC and PCC

	Positive (*N *= 23)	Negative (*N *= 332)	*P* value
Sex			
Male	19	225	0.167
Female	4	107	
Age			
> 65	6	184	0.009
≤ 65	17	148	
Tested tumor specimen			
Resection	7	118	0.822
Biopsy	16	214	
Percutaneous biopsy	13	164	
EUS-FNA	2	24	
Endoscopic biopsy	1	26	
Stage			
Metastasis	13	168	0.124
Recurrence	8	77	
Locally advanced	2	87	
Differentiation			
Poorly	1	61	0.178
Moderate/well	9	129	
Unknown	13	142	
Primary site			
ICC	20	252	0.311
PCC	3	80	
Macroscopic type^a^			
Mass forming	20	219	0.221
Periductal infiltrating	0	27	
Intraductal growth	0	1	
Unknown	0	5	
Smoking history			
Positive	17	204	0.372
Negative	6	122	
Unknown	0	6	
Alcohol abuse (ethanol/day)			
≥ 60 g	7	38	0.060
< 60 g	16	289	
Unknown	0	5	
HCV Ab and/or HBs Ag			
Either positive	6	30	0.020
Others	17	302	
Pancreaticobiliary maljunction			
+	0	1	1.0
−	23	331	
PSC			
Positive	0	1	1.0
Negative	23	331	
Printing industry			
Positive	0	3	1.0
Negative	23	317	
Unknown	0	12	
Cholelithiasis			
+	1	21	1.0
−	22	311	
Family history about malignant tumor			
+	11	172	0.830
−	12	160	

*EUS-FNA* endoscopic ultrasound-fine needle aspiration, *PSC* primary sclerosing cholangitis

^a^For macroscopic type, only ICC was analyzed

**Table 4:** Multivariate analysis of *FGFR2*-positive patients (logistic regression analysis)

Multivariate analyses	Odds ratio	95% CI	*P* value
Age			
≤ 65 / > 65	3.20	(1.22 − 8.41)	0.0181
HCV Ab and/or HBs Ag			
** + /** −	2.98	(1.07 − 8.28)	0.0365
Alcohol abuse (ethanol/day)			
≥ 60 g/ < 60 g	1.32	(0.42− 1.81)	0.0702

**Fig. 2:**
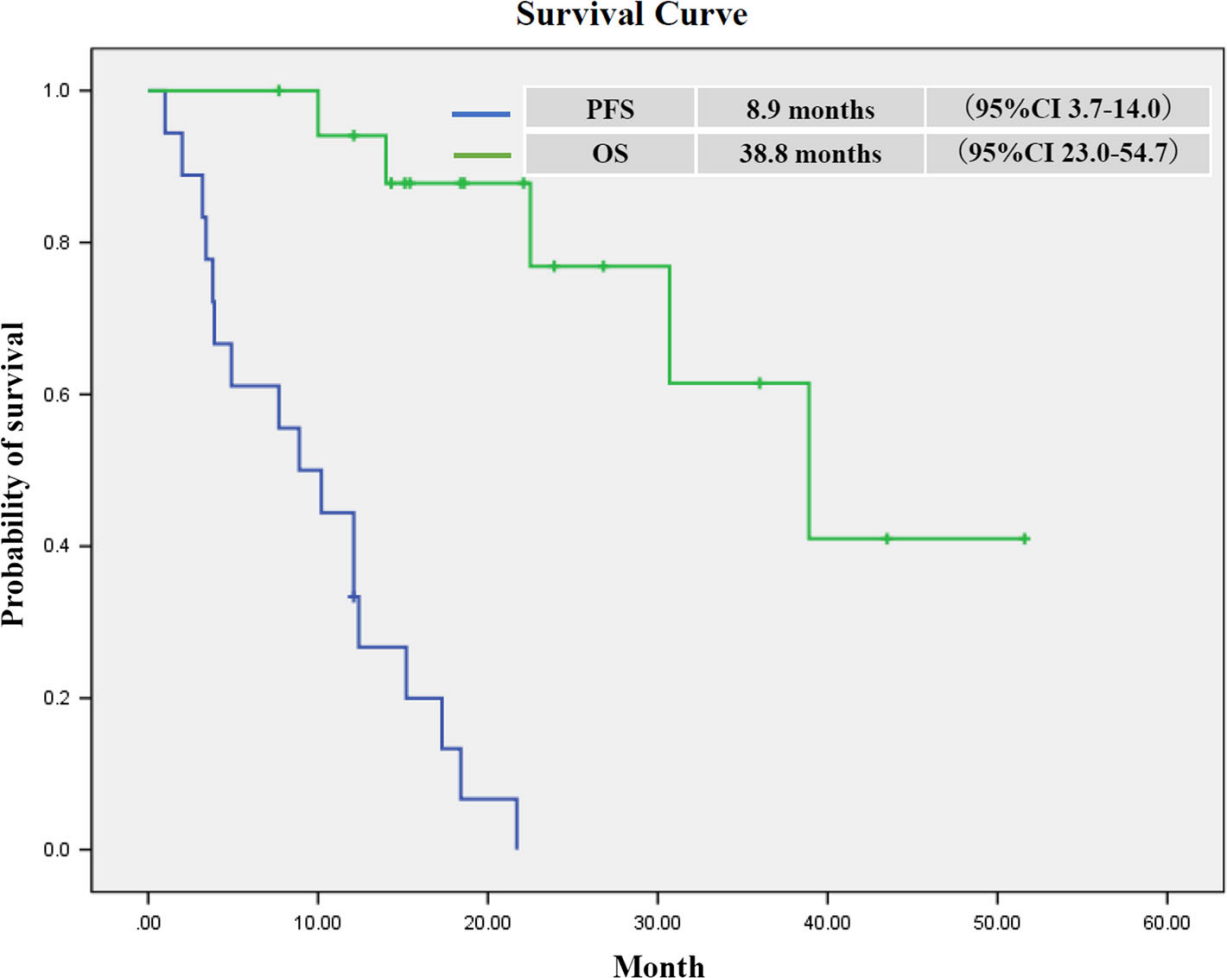
Overall survival (OS) curve for *FGFR2* rearrangement-positive cases in the second period, and progression-free survival (PFS) curve for first-line chemotherapy

In the second period, we investigated the details and clinical responses of first-line chemotherapy regimens for cases positive for *FGFR2* rearrangements. The gemcitabine and cisplatin (GC) therapy was performed in 17/18 patients, and gemcitabine + S-1 therapy was performed in the remaining patient. The response rate and disease control rate of the first-line treatment were 22% (4/18) and 66.7% (12/18), respectively. The overall survival (OS), from the initiation of first-line chemotherapy, of *FGFR2* rearrangement-positive patients is shown in Fig. 2. The median OS was 38.8 months for the 18 *FGFR2* rearrangement-positive patients, 13 of whom received molecular targeting therapy with FGFR inhibitors.

## Discussion

*FGFR2* gene alterations were reported to be associated with early stages [17]; however, no study has selectively examined advanced cases. In this study, the frequency of *FGFR2* rearrangement-positive cases was 7.4% among patients with advanced/recurrent ICC, which was lower than those of previous reports (13–14%) that analyzed surgically resected cases [12, 14]. In contrast, the current study and previous reports [13] that targeted advanced/recurrent patients indicated lower frequency (7.4–9%). Churi et al. suggested that the activation of the FGF/FGFR pathway associates with better prognosis in patients with ICC [18]. Hayashi et al. reported that *FGFR2* fusion gene-positive ICC develops from peripheral bile ducts and is found in type 2, which was characterized by low mucin production, mass forming macroscopic type and better prognosis [19]. The different frequency of *FGFR2* rearrangements between patients undergoing resection, and those with advanced cancer, could be due to the preferential occurrence of *FGFR2* rearrangements in patients with peripheral and mass forming type, which progress slowly and are frequently treated by surgical resection. This study also revealed that 3.6% of patients with PCC had *FGFR2* rearrangements. In clinical practice, ICC and PCC are often difficult to discriminate.

Additionally, in the current study, three PCC patients were found to be *FGFR2* rearrangement positive, two of which had intrahepatic extension. However, these two cases were resected and the diagnosis of the primary region were made via detailed pathological assessment of resected specimen. The remaining one case was diagnosed by ERCP, enhanced CT and biopsy, and was determined to have a perihilar lesion without mass formation of intrahepatic extension. Hence, the diagnosis of the primary region as “perihilar bile duct” for these three cases is reliable. Our results indicate that PCC could also be screened for *FGFR2* rearrangements as a potential target category of FGFR inhibitors.

According to the post hoc targeted RNA sequencing using FFPE specimens, the sensitivity and specificity of the FISH assay to detect *FGFR2* rearrangements was 100% and 99.0%, respectively, indicating that FISH is a reliable diagnostic assay method. Meanwhile, a recent report indicated that multiplex NGS panel testing is possible even with FNA samples, although it did not survey any *FGFR2* fusions [20]. NGS is a useful and convenient method. However, there are also associated concerns regarding sample preparation, including the amount of DNA required (sufficient for tumor cell count and percent tumor content), sample storage time, and probe design according to the partner gene. Additionally, NGS requires longer turnaround time than the FISH technique. Therefore, the complementary use of several techniques including NGS and FISH is desirable in clinical settings.

Besides *FGFR2* in-frame fusions, recurrent C-terminal truncation events translocating *FGFR2*, without its 3’-UTR, to intergenic regions were also reported in ICC [21]. ICC patients with 3’-UTR truncated *FGFR2* transcripts exhibited higher RNA expression compared to wild-type *FGFR2* transcripts. Meanwhile, C-terminal truncation of FGFR2 showed transforming ability in gastric cancer [22]. As target RNA sequencing in this study analyzed only coding exon sequences, no structural information was available regarding the 3’-UTR of *FGFR2*. Nevertheless, our data revealed that 4/6 FISH-positive cases exhibiting loss of the 3’-probe (YG signal) had no *FGFR2* fusion transcript, which may reflect the 3’-UTR loss of *FGFR2*. Further analysis will clarify whether clinical responses to FGFR-targeted therapy differ depending on rearrangement pattern on FISH.

Over 40 genes have been detected as *FGFR2* fusion partners in ICC [17, 23]. Although break-apart FISH assays cannot identify the fusion partner gene, it can clarify the presence/absence of *FGFR2* rearrangements (fusions/truncations) including those involved with unknown partners. All *FGFR2* fusion-positive cases showed YGR type in this FISH analysis, and samples showing YG type included both fusion and truncation. Therefore, FISH alone analysis can distinguish fusion from fusion or truncation if the FISH type is YGR. YR FISH type was rare, and treated as *FGFR2* rearrangement ‘negative’ in this study. Besides case 25, two case showed YR FISH type (11% and 23%) with low *FGFR2* rearrangement-positive signal (YGR or YG: 5% and 0%, respectively). YR type denotes 5ʹ probe deletion of *FGFR2* and might lose oncogenic driver activity. Then we think YR FISH type is incidental and happens rare.

Break-apart *ALK* FISH assay was approved by the FDA as a companion diagnostic for detecting *ALK* rearrangements in lung cancer patients who may benefit from treatment of ALK tyrosine kinase inhibitor therapy using ≥ 15% as a cutoff value [24]. In meta-analysis of *ALK* rearrangement-positive non-small lung cancer, higher percentage of *ALK* rearrangement-positive cells tend to respond better to the crizotinib therapy [25]. At present, our analysis cannot judge whether the percentage of FISH-positive cell rate influence the outcome because of the limited cases. In this study, we preset the cutoff value as ≥ 7% for a positive FISH result. This threshold was obtained from the assay background (6.4%, mean + 2SD) in the analysis of a limited number of genotyped *FGFR2* fusion-positive and -negative controls from surgically resected specimens. However, this ≥ 7% cutoff might be too low for the small biopsy samples. In two fusion transcript-negative cases (case 24, 25), the FISH-positive cell rate (8%, 9%) was close to the ≥ 7% preset cutoff value, and were thus treated as false positives after RNA sequencing. Hence, further optimization of diagnostic thresholds should be necessary, such as setting a higher cutoff value, as FISH-positive cell rate in all cases was higher than 15%, except for the two fusion transcript-negative cases.

*FGFR2* rearrangement was more frequently detected in younger and hepatitis virus-positive patients. A previous study analyzing somatic mutations related to ICC with cirrhotic liver reported higher frequencies of either *IDH* mutations or *FGFR2* alterations [26]. Both that report and our study suggest a possible association with a background of continuous damage to hepatocytes, such as those related to virus infection [27, 28] and the presence of *FGFR2* rearrangements.

In *FGFR2* rearrangement-positive 18 patients, the median OS was 38.8 months, and they have good prognosis as survival in advanced/recurrent ICC/PCC. It may be due to the clinical feature of *FGFR2* fusion/rearrangement ICC/PCC patients [18] and therapeutic intervention of FGFR inhibitors. Among them, 17 patients received combination therapy of GC as first-line chemotherapy, and 1 patient received combination therapy of gemcitabine and S-1. The median progression-free survival (PFS) of these 18 patients in first-line chemotherapy was 8.9 months (Fig. 2). Valle et al. reported that the median PFS of GC therapy was 8.0 months in the ABC-02 trial [3]. The result of PFS in this study was similar to the previous reports. Therefore, we consider that a crucial factor for good prognosis in these 18 patients was treatment with FGFR inhibitors, as was reported in 13 of the 18 patients (72%).

Certain limitations were noted in this study. First, since the number of positive patients was small, the prognostic significance of *FGFR2* rearrangement positivity must be confirmed in larger studies. Second, since clinical trials of FGFR inhibitors, including our cases, are ongoing, the clinical utility of our FISH assay as a companion diagnostic test to predict the therapeutic efficacy of FGFR inhibitors requires further evaluation in the future.

In conclusion, this study demonstrated the feasibility of *FGFR2*-FISH assay using biopsy specimens of BTC, and showed that 7.4% of cases in advanced/recurrent ICC retained *FGFR2* rearrangements, and 3.6% cases in advanced/recurrent PCC also carried the alterations. Hence, this accounts for the first report of positive PCC cases. Moreover, younger age and a history of hepatitis viral infection are associated with the presence of *FGFR2* rearrangements. These findings have important implications for elucidating the pathophysiology of *FGFR2* rearrangements, and will be useful in developing targeted therapy for ICC and PCC.

## Electronic supplementary material

Below is the link to the electronic supplementary material.

Supplementary file1 (TIFF 4381 kb)**Supplementary Figure S1.** Representative FISH images from *FGFR2* rearrangement-positive/negative cases. Break-apart FISH assay revealed the rearrangements with the 5ʹ-probe of *FGFR2* shown in green and the 3ʹ-probe shown in red. The rearrangement positive cells were defined as having any split signal (YGR type) as in (a) or any isolated green signal (YG type) as in (b), while any isolated red signal (YR type) as in (c) was treated as rearrangement-negative. (d) Schematic diagram of *FGFR2* FISH probes and FISH patterns. Thin arrow, green signal; bold arrow, red signal; arrowhead, yellow signal

Supplementary file2 (PDF 42 kb)
