# Supplementary figures and images for: Molecular detection and clinicopathological characteristics of advanced/recurrent biliary tract carcinomas harboring the FGFR2 rearrangements: a prospective observational study (PRELUDE Study)
Source: J Gastroenterol. 2020 Oct 26;56(3):250–60. doi: 10.1007/s00535-020-01735-2 (PMC7932978; doi:10.1007/s00535-020-01735-2)

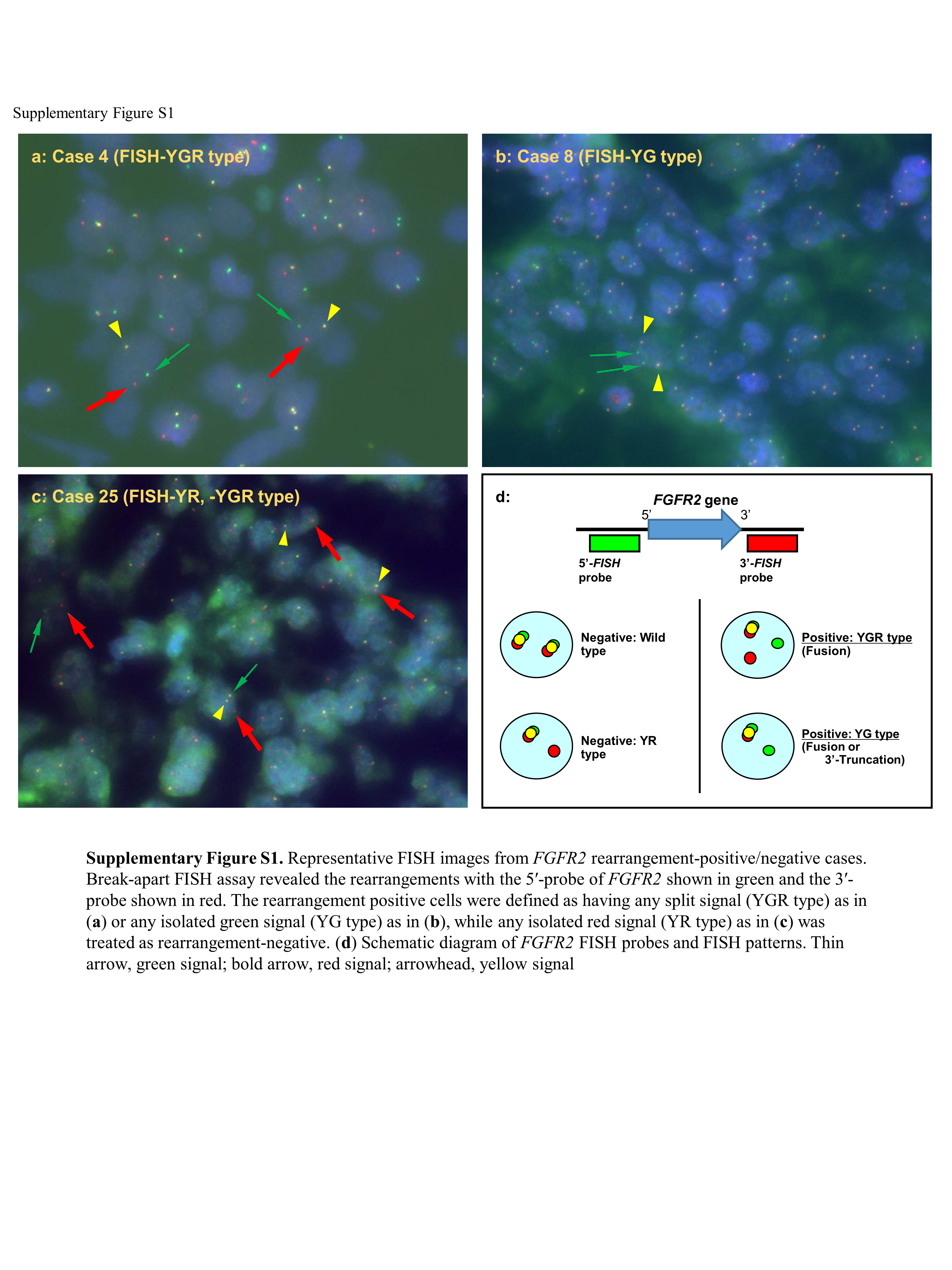

Supplement: Supplementary file 1 — Supplementary file1 (TIFF 4381 kb)Supplementary Figure S1. Representative FISH images from FGFR2 rearrangement-positive/negative cases. Break-apart FISH assay revealed the rearrangements with the 5ʹ-probe of FGFR2 shown in green and the 3ʹ-probe shown in red. The rearrangement positive cells were defined as having any split signal (YGR type) as in (a) or any isolated green signal (YG type) as in (b), while any isolated red signal (YR type) as in (c) was treated as rearrangement-negative. (d) Schematic diagram of FGFR2 FISH probes and FISH patterns. Thin arrow, green signal; bold arrow, red signal; arrowhead, yellow signal [file 535_2020_1735_MOESM1_ESM.tif]
