# Supplementary material for: Molecular detection and clinicopathological characteristics of advanced/recurrent biliary tract carcinomas harboring the FGFR2 rearrangements: a prospective observational study (PRELUDE Study)
Source: J Gastroenterol. 2020 Oct 26;56(3):250–60. doi: 10.1007/s00535-020-01735-2 (PMC7932978; doi:10.1007/s00535-020-01735-2)
Supplement: Supplementary file 2 — Supplementary file2 (PDF 42 kb) [file 535_2020_1735_MOESM2_ESM.pdf]

Supplementary Figure S2

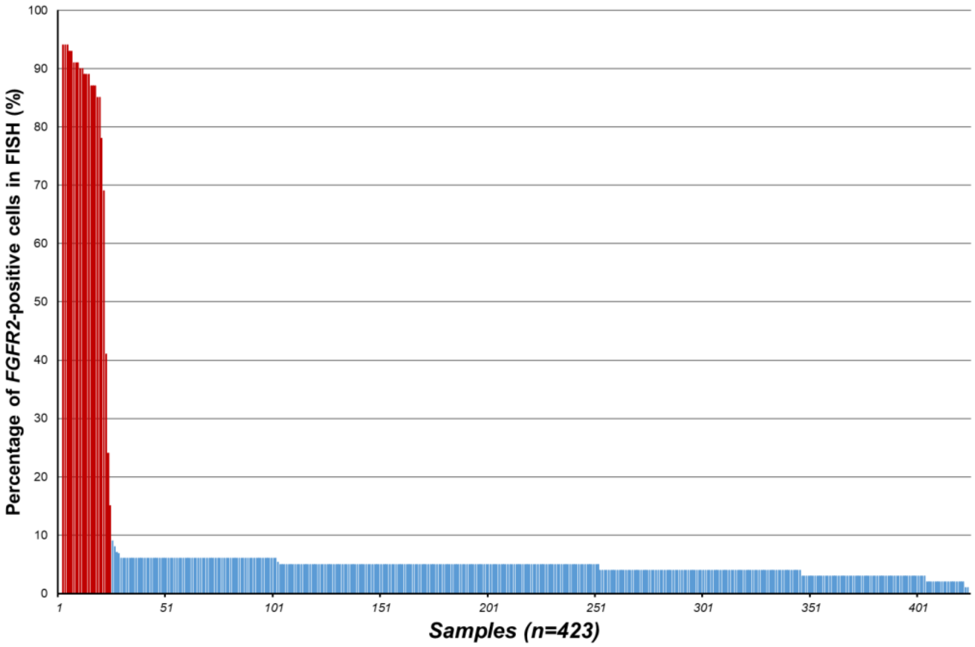

Supplementary Figure S2. Distribution of the percentages of *FGFR2*-positive cells in FISH analysis. Samples (nos. 1-23, median 89%, range 15-94%) validated with targeted RNA sequencing were shown in red. A total of 400 patients were *FGFR2* rearrangement negative (median 5%, range 0-9%), shown in blue.
